# *De novo* Assembly and Characterization of the Transcriptome of Broomcorn Millet (*Panicum miliaceum* L.) for Gene Discovery and Marker Development

**DOI:** 10.3389/fpls.2016.01083

**Published:** 2016-07-21

**Authors:** Hong Yue, Le Wang, Hui Liu, Wenjie Yue, Xianghong Du, Weining Song, Xiaojun Nie

**Affiliations:** ^1^College of Agronomy, Northwest A&F UniversityYangling, China; ^2^State Key Laboratory of Crop Stress Biology in Arid Areas, Northwest A&F UniversityYangling, China; ^3^Australia-China Joint Research Centre for Abiotic and Biotic Stress Management in Agriculture, Horticulture and Forestry, Northwest A&F UniversityYangling, China

**Keywords:** abiotic stress, broomcorn millet, transcriptome, qRT-PCR, SSR

## Abstract

Broomcorn millet (*Panicum miliaceum* L.) is one of the world’s oldest cultivated cereals, which is well-adapted to extreme environments such as drought, heat, and salinity with an efficient C4 carbon fixation. Discovery and identification of genes involved in these processes will provide valuable information to improve the crop for meeting the challenge of global climate change. However, the lack of genetic resources and genomic information make gene discovery and molecular mechanism studies very difficult. Here, we sequenced and assembled the transcriptome of broomcorn millet using Illumina sequencing technology. After sequencing, a total of 45,406,730 and 51,160,820 clean paired-end reads were obtained for two genotypes Yumi No. 2 and Yumi No. 3. These reads were mixed and then assembled into 113,643 unigenes, with the length ranging from 351 to 15,691 bp, of which 62,543 contings could be assigned to 315 gene ontology (GO) categories. Cluster of orthologous groups and Kyoto Encyclopedia of Genes and Genomes (KEGG) analyses assigned could map 15,514 unigenes into 202 KEGG pathways and 51,020 unigenes to 25 COG categories, respectively. Furthermore, 35,216 simple sequence repeats (SSRs) were identified in 27,055 unigene sequences, of which trinucleotides were the most abundant repeat unit, accounting for 66.72% of SSRs. In addition, 292 differentially expressed genes were identified between the two genotypes, which were significantly enriched in 88 GO terms and 12 KEGG pathways. Finally, the expression patterns of four selected transcripts were validated through quantitative reverse transcription polymerase chain reaction analysis. Our study for the first time sequenced and assembled the transcriptome of broomcorn millet, which not only provided a rich sequence resource for gene discovery and marker development in this important crop, but will also facilitate the further investigation of the molecular mechanism of its favored agronomic traits and beyond.

## Introduction

Broomcorn millet (*Panicum miliaceum* L.) is one of the earliest domesticated cereals worldwide with historical, evolutionary and agricultural significance (Yang et al., 2012; Stephens et al., 2014). It has been revealed that Broomcorn millet was domesticated as early as 10,000 years ago in the semiarid regions of China (Lu et al., 2009), and it was an indispensable food staple in many semiarid regions of East Asia before the cultivation of rice and wheat (Hu et al., 2008). Thus, Broomcorn millet played a vital impact on human civilization (Crawford, 2006; Bellwood et al., 2007). Genetically, Broomcorn millet is a tetraploid species with a chromosome number of 36 (2N = 4X = 36; Hunt et al., 2010). As a short-day C4 crop, it has many favored agronomic traits, such as high productivity yield, short growing season (60–90 days), a lower water requirement and is well-adapted to extreme conditions (Hunt et al., 2014). Previous studies revealed that Broomcorn millet showed high drought and salinity tolerance (Dai et al., 2011). Thus, discovery and identification of the stress-responsive genes from broomcorn millet will not only provide useful information for better understanding the molecular mechanism of stress tolerance, but also provide indispensable gene resources for tolerance improvement in this species as well as other crops (Jiang and Deyholos, 2006).

With the development of next generation sequence (NGS) technology, RNA sequencing (RNA-seq) has gradually become a powerful and high-efficiency method to obtain a large number of transcripts and identify differentially expressed genes (DEGs) at the transcriptome level, which has been widely used in various plants (Clark et al., 2015; Sartelet et al., 2015; Xu et al., 2015; Zhan et al., 2015). Prior to this study, transcriptome analysis has not been performed in broomcorn millet, which has limited further gene identification and molecular mechanism studies (Miller et al., 2002; Salgado et al., 2014; Yates et al., 2014). Here, *de novo* assembly and analysis of the broomcorn millet transcriptome was performed using High-throughput Illumina paired-end RNA sequencing technology, with the purpose to enrich the genetic information and sequence resources for facilitating gene discovery and marker development studies. This is the first study to report the transcriptome characteristics of broomcorn millet, which will provide the useful information for molecular studies, and also shed light on the molecular mechanism of stress tolerance in Broomcorn millet.

## Materials and Methods

### Plant Materials and RNA Extraction

Two Broomcorn millet genotypes, namely Yumi No. 2 and Yumi No. 3, which were kindly provided by Dr. Bai-Li Feng, College of Agriculture, Northwest A&F University, were used as materials in this study. Yumi No. 2 is a waxy, drought-sensitive cultivar, while Yumi No. 3 is a non-waxy, drought and salt tolerant cultivar. Seeds of both cultivars were germinated and grown in pots containing peat mixed sand at a 1:1 ratio. Plants were normally watered and grown under glasshouse conditions (22°C, 16 h photoperiod/20°C, 8 h dark period). For RNA-Seq library construction, tissue samples including leaves, stems, spikes, and roots were collected from five plant individuals grown at different time points. Leaves and roots were harvested at the seedling, jointing, booting, and filling stages while stems were harvested at the jointing, booting, filling, and mature stages. Spikes were harvested at the mature stages. Three independent tissue samples collection were performed as biological replicates. All the samples were immediately frozen in liquid nitrogen and stored at -80°C for RNA isolation.

Total RNA from collected samples was separately isolated using TRIzol reagent (Invitrogen) according to the manufacturer’s instructions. Then, the quality of RNA was checked by agarose gel electrophoresis and Agilent 2100 Bioanalyzer (Agilent Technologies, Santa Clara, CA, USA) and the quantity was checked by NanoDropND-1000 Spectrophotometer (NanoDrop Technologies, Wilmington, DE, USA). Finally, the equal amounts of RNA isolated from Yumi No. 2 and Yumi No. 3 tissues were pooled together into a single RNA sample respectively, and then the pooled RNA sample was used for RNA sequencing.

### Library Construction and Illumina Sequencing

The RNA-seq library construction and sequencing were performed using the Illumina’s standard pipeline (Illumina, San Diego, CA, USA). In brief, magnetic oligospheres were used to remove rRNA or tRNA and enrich mRNA. mRNA was further sheared into short fragments of 180 bp in size were recovered and column purified, then subjected to first strand cDNA synthesis using random hexamer-primed synthesis of first-strand cDNA, followed by second-strand cDNA synthesis using DNA polymerase I and RNase H (Invitrogen). The cDNA fragments were end-repaired and A-tailed, and then index adapters were ligated. After the purified cDNA libraries were amplified by polymerase chain reaction (PCR) for 15 cycles and PCR products were separated by Certified Low Range Ultra Agarose, the suitable fragments were selected for deep sequencing. Sequencing was performed on the IlluminaHiSeq2500 platform with the PE100 approach by Shanghai Majorbio Bio-pharm Technology Corporation. All clean Illumina sequencing data have been deposited in SRA database with the accession number of SAMN05255231.

### Sequence Data Analysis and *De novo* Assembly

The quality of the raw reads of two libraries was checked using SeqPrep and Sickle software. Adapter contamination, low quality bases (≤ 20), reads containing more than 10% ambiguous bases, and reads containing less than 20 bases were removed. The obtained clean reads were mixed and then *de novo* assembled using the Trinity program with the default parameters^1^. K was set to 25 for inchworm analysis and the transcript isoform with the highest relative abundance after butterfly analysis was selected as the representative transcript for each gene. Clustering analysis with CD-HIT software, with 95% identity, was performed to reduce redundancy from transcripts derived from homeologous genes or different alleles of the same genes, which are common artifacts of pooling tissues from multiple individuals for transcrpitome sequencing.

### Functional Annotation

To determine the predicted function, all unigenes were used as blastx queries against the following databases with an e-value < 1e-5, including NCBI Non-redundant Protein (NR) database (2016/1/12), Swiss-Prot protein database (UniProtKB/Swiss-Prot protein knowledgebase release 2015_12 statistics) and Cluster of Orthologous Groups database (COG) as well as Kyoto Encyclopedia of Genes and Genomes pathway database (KEGG). To evaluated sequences similarity and predicted function according to homologous genes, three close relative species of broomcorn millet, including *Panicum halli, Panicum virgatum* and *Setaria italica*, were used to analyze. The genome annotaion sequences of *P. halli*^2^ and *P. virgatum*^3^ were obtained from JGI phytozome. The genome annotaion sequences of *S. italica* were obtained from the Foxtail millet Database^4^. The hits with the highest sequence similarity were retrieved for analysis. Based on NR annotation, 10 top-hit species were identified and gene ontology (GO) classifications were annotated by the Blast2GO program. KEGG produced annotation of metabolic pathways. Goatools^5^ and KOBAS (Xie et al., 2011) was used to identify enriched GO and KEGG in the DEGs between Yumi No. 2 and Yumi No. 3. DEGs were identified using Fisher’s exact test and *P*-values were corrected for multiple hypothesis testing.

### SSRs and SNPs Markers Screening

Potential simple sequence repeats (SSRs) were detected using MISA software^6^. In this study, repeats of one to six nucleotides in length were considered. The minimum reiterations units were 10 repeat units for mononucleotides, six for dinucleotides, and four for tri-, tetra-, penta-, and hexa-nucelotides. The maximal distance was 100 nucleotides interrupting two SSRs in a compound microsatellite. Assembled contigs were scanned for single nucleotide polymorphisms (SNPs) with SNP detection software SOAPsnp (Li et al., 2009) using the method as described by Chen et al. (2014), which has been used for SNP calling in *Stevia rebaudiana* with low quality mapping scores (≤ 20) and less than five nucleotides apart were discarded.

### Analysis and Annotation Differentially Expressed Genes (DEGs)

Bowtie was used to perform the read mapping. The number of reads per kilobase of exon region per million mapped reads (RPKM) was used to normalize the expression values of reads. Raw read counts were utilized with edgeR^7^ to identify DEGs. In multiple hypothesis testing, false discovery rate (FDR) was used to select the threshold *P*-value (Benjamini et al., 2001). An FDR ≤ 0.05 and fold change (FC) ratio larger than 2 (| log_2_ FC|≥ 1) were chosen in our study to determine the DEGs. Scatter and volcano plots were drawn by geWorkbench platform^8^ using the value of logRPKM and log_2_FC. All DEGs were subjected to GO and KEGG annotation and cluster analysis of DEG patterns was performed using hCluster software^9^ following the method previously described by Liu G. et al. (2012).

### qRT-PCR Analysis

First, Broomcorn millet plants, including Yumi No. 2 and Yumi No. 3, were adequately watered and grown under glasshouse conditions (22°C, 16 h photoperiod/20°C, 8 h dark period). After grown for 1 month, the plants were transferred into 4C, 38°C or 200 mM NaCl, which represented low temperature, heat and salt treatment, respectively (Li et al., 2014). Whole plants were separately collected at 0, 3, 6, 12, and 24 h under above treatment as well as under no stress treatment as control. The experiment was conducted three independent times with five biological replicate seedlings per treatment. Tissue from the five replicate samples was pooled for RNA isolation using the RNAiso reagent (TaKaRa, Japan) and the three experiments were treated as biological replicates for the RNA extraction, and then equivalent amounts of RNA from three biological replicates of each sample was pooled into a single RNA. cDNA was prepared by using the PrimeScriptTM RT reagent Kit (TaKaRa, Japan) following the manufacturer’s instructions. Quantitative reverse transcription polymerase chain reaction (qRT-PCR) was analyzed using SYBR Green master mix (TaKaRa, Japan) and ABI 7300 real-time PCR system (Applied Biosystems, USA). The thermal cycling conditions were as follows: 3 min at 94°C, followed by 40 cycles each consisting of 95°C for 15 s, 60°C for 30 s, 72°C for 1 min. *Actin* was used as an internal control (Supplementary Table S1). Each reaction was performed in triplicate and the2^-ΔΔCt^ method was used to calculate the expression levels. Student’s *t*-test was used to statistics analyze.

## Results and Discussion

### Transcriptome Sequencing and *De novo* Assembly

**FIGURE 1:**
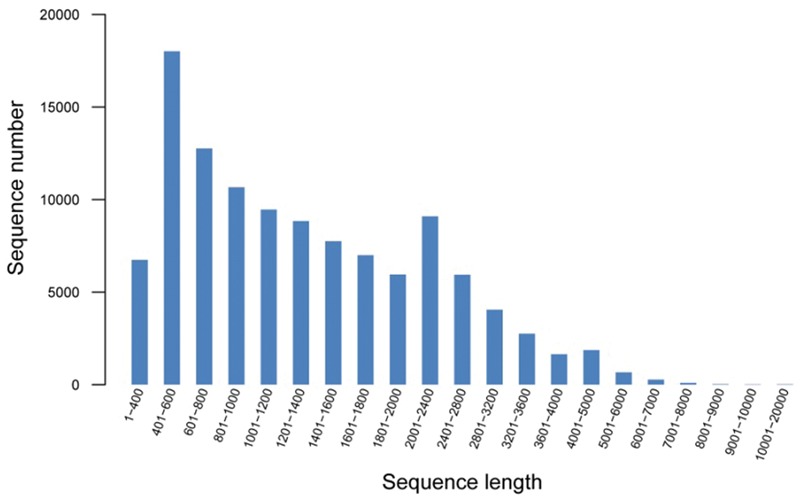
**Size distribution of all assembled unigenes using the combined reads of common millet cultivars Yumi No. 2 and Yumi No. 3**.

To comprehensively generate a broomcorn millet transcriptome, the RNA isolated from leaves, stem, root, shoots, flower, and spike of two cultivars were equally pooled and sequenced separately using the Illumina Hiseq2000 platform. After sequencing and quality filtering, a total of 45,406,730 high-quality reads for Yumi No. 2 and 51,160,820 reads for Yumi No. 3 were obtained, accounting for approximately 4.3 and 4.9 Gb, respectively (Supplementary Table S2). Then, the generated reads were mixed and *de novo* assembled using Trinity software. A total of 113,643 contigs with a total assembly length of 164,535,293 bp were obtained. To investigate the quality and coverage of assembly, we then mapped the paired reads of the two cultivars back to these contigs. Results showed that 132,599,016 (93.80%) and 113,424,962 (93.29%) reads belonging to Yumi No. 2 and Yumi No. 3 respectively could be mapped back to the contigs, supporting the accuracy of the assembly. Further analysis found that the size distribution of these contigs ranged from 351 to 15,691 bp with an average size of 1,448 bp, of which 24,750 (21.78%) ranged from 100 to 600 bp, and 23,423 (20.61%) ranged from 600 to 1000 bp; 65,470 unigenes with lengths longer than 1 kb were identified (**Figure 1**). To the best of our knowledge, the sequences of broomcorn millet are deficient at present (Hunt et al., 2011). Therefore, the unigene dataset reported here significantly enriches the sequence resources and genetic information of broomcorn millet, which provides the foundation for further study of gene expression, gene function and gene regulation pathways in broomcorn millet.

### Annotation and Functional Characterization of the Broomcorn Millet Transcriptome

**FIGURE 2:**
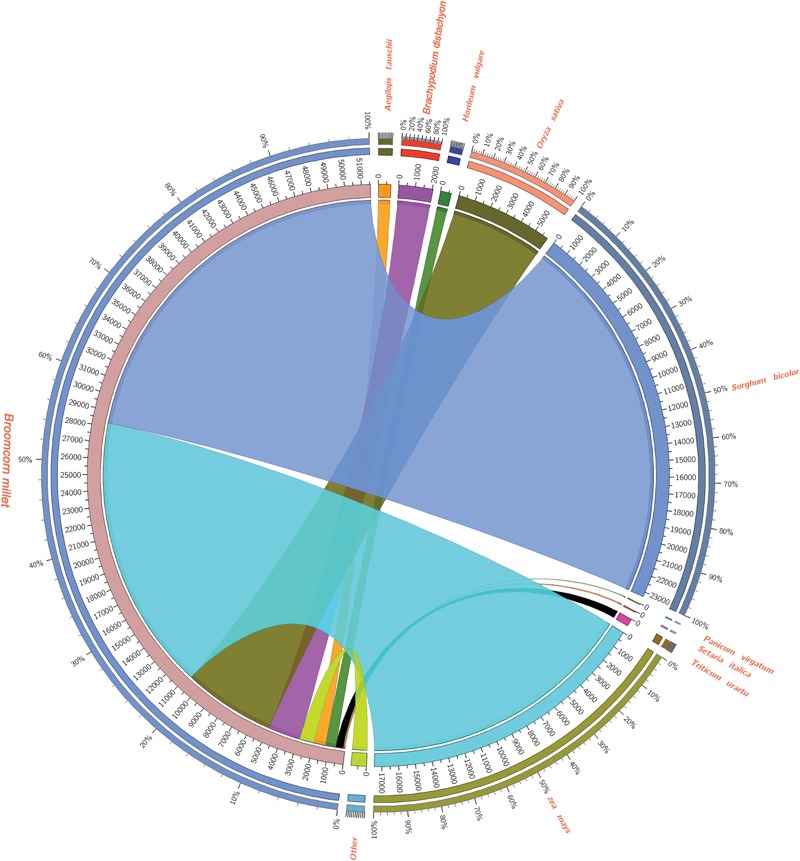
**Summary of Broomcorn millet unigenes similarity search compared to other monocots using Circos (http://circos.ca/).** 43.68% unigenes of broomcorn millet have homologous matches to *Sorghum bicolor* transcripts, 34.74% have hits to *Zea mays*, 11.19% have hits to *Oryza sativa*, 3.65% have hits to *Brachypodium distachyon*, 1.52% have hits to *Hordeum vulgare*, 1.45% have hits to *Aegilops tauschii*, 0.90% have hits to *Triticum urartu*, 0.14% have hits to *Setaria italica*, and 0.09% have hits to *Panicum virgatum* as well as 1.78% have hits to other species, respectively.

In order to assess and annotate the assembled unigenes, 113,643 assembled unigenes generated by Trinity software were subjected to blastx similarity searches against NCBI’s NR database, Swiss-Prot, COG, KEGG of proteins with an e-value cut off of 10^-5^. As a result, 51,629 unigenes had matches to proteins in the NR database, and 40,407 unigenes were similar to proteins in the Swiss-Prot database. Totally, 60,352 unigenes were profitably annotated in Nr, Swiss-Prot, GO, COG, and KEGG. The size distribution of these open reading frames was shown in Supplementary Figure S1. Furthermore, we examined the homologs of broomcorn millet unigenes with other monocot species, including *Sorghum bicolor, Oryza sativa, Brachypodium distachyon, Hordeum vulgare, Aegilops tauschii, Triticum urartu, P. virgatum, S. italica* by protein similarity search in NCBI NR database. Results showed that 35,888 (43.68%) unigenes of broomcorn millet have homologous matches to *S. bicolor* transcripts, 28,537 (34.74%) have hits to *Zea mays*, 9,194 (11.19%) have hits to *O. sativa*, 2,995 (3.65%) have hits to *B. distachyon*, 1,247 (1.52%) have hits to *H. vulgare*, 1,190 (1.45%) have hits to *A. tauschii*, 742 (0.90%) have hits to *Triticum urartu*,73(0.14%) have hits to *S. italica*, and 50 (0.09%) have hits to *P. virgatum* as well as 921 (1.78%) have hits to other species, respectively (**Figure 2**). It is unexpected that only a few Broomcorn millet unigenes showed homologous significantly matches to *S. italica* and *P. virgatum*. Further analysis found this might be as the result of the best hits with better e-values and similarity distributions in *S. bicolor, Zea mays*, and *O. sativa* compared to *P. halli, P. virgatum*, and *S. italica.* To further evaluate accurately sequences similarity with *P. halli, P. virgatum* and *S. italica*, the predicted protein sequences of these three species were used to identify the homologs in broomcorn millet unigenes by blastx search with an e-value cutoff of 10^-5^. Results showed that 88,538 (77.91%) unigenes of broomcorn millet have homologs hits to *P. halli* genome annotations, 90,229 (79.40%) had hits to *P. virgatum* and 84,024 (73.94%) had hits to *S. italica*, respectively. At the same time, e-value and similarity distribution through against Nr databases, *P. halli, P. virgatum*, and *S. italica* genome annotations was shown in Supplementary Figure S2.

**FIGURE 3:**
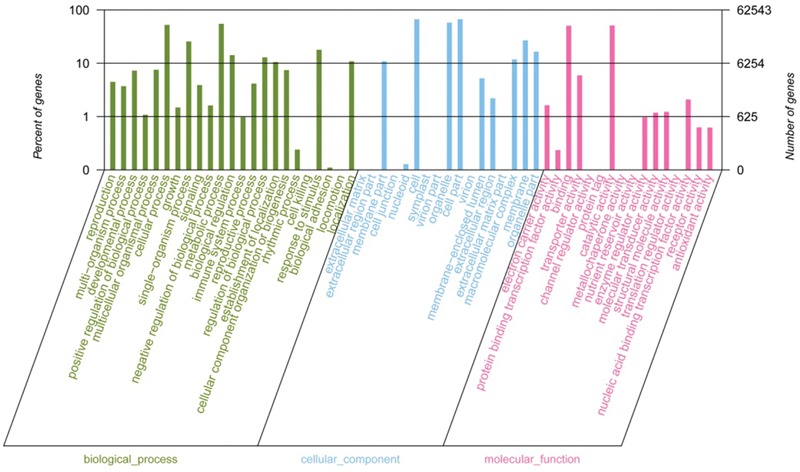
**Level 2 GO annotations using the gene ontology (GO) of assembled transcripts.** The results are classified into biological process, cellular component and molecular function.

To preliminarily understand the function of the broomcorn millet unigenes, those with homologs to previously annotated sequences in the NR database were further annotated with GO terms using the Blast2GO tool (Conesa et al., 2005). A total of 62,543 unigene sequences were annotated to three major GO classes. The largest class was cellular component, accounting for 42.47% of the total annotated unigenes, followed by biological process (38.93%) and molecular function (18.60%). Within the cellular component, ‘cell,’ ‘cell part,’ and ‘organelle’ were the most abundant among the 56 categories, which together accounted for 72.14% of the genes belonging to this class. In biological process, ‘metabolic’ and ‘cellular processes’ were the largest and second largest categories, accounting for 44.09% of the sequences assigned to this term. In 16 different molecular function categories, the two most abundant categories were ‘catalytic activity’ and ‘binding,’ which accounted for 87.44% for the genes of this class (**Figure 3**).

**FIGURE 4:**
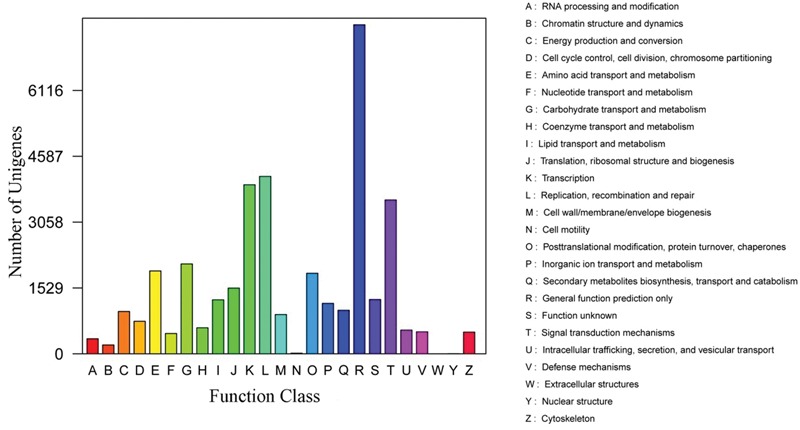
**Eukaryotic cluster ortholog groups classification of assembled transcripts**.

Furthermore, the assembled unigenes were aligned to the KOG database to classify their putative function. Result showed that a total of 33,671 unigenes could be matched to genes in the KOG database. These unigenes were classified into 25 different functional classes. Among transcripts with matches to the KOG database, ‘the function prediction only class’ (7,071, 21.00%) represented the largest group, followed by ‘signal transduction mechanisms’ (4,986; 14.81%), ‘post-translational modification, protein turnover and chaperones’ (3,835; 11.39%) ‘transcription’ (2,570; 7.63%), while only a few unigenes were assigned to ‘extracellular structures’ (70; 0.21%) and ‘cell motility’ (11; 0.03%; **Figure 4**).

Finally, KEGG analysis was used to assigned unigenes to metabolic pathways. 15,514 unigenes were mapped to 202 KEGG pathways. Among them, the most represented pathways were metabolic pathways (ko01100; 25.65%), followed by biosynthesis of secondary metabolites (ko01110; 10.71%), biosynthesis of amino acids (ko01230; 3.57%), pyrimidine metabolism (ko00240; 2.75%), purine metabolism (ko00230; 2.70%), peroxisome (ko04146; 2.55%), and spliceosome (ko03040; 2.50%) as well as plant–pathogen interaction (ko05169; 2.37%; Supplementary Figure S3 and Supplementary Table S3).

### Identification of SSRs and SNPs Loci in Broomcorn Millet

**FIGURE 5:**
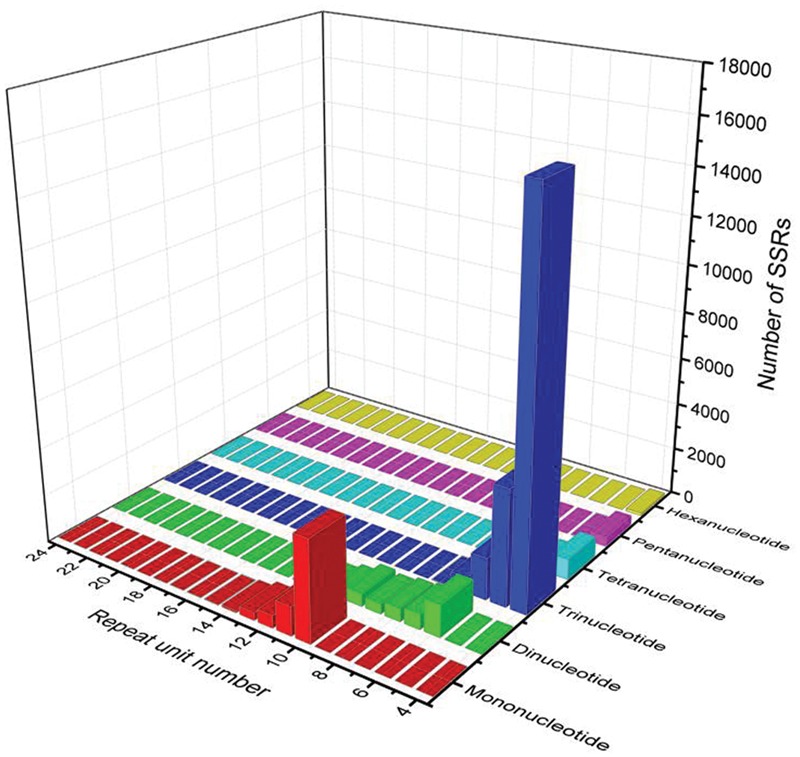
**Number of different SSRs repeat types between Yumi No. 2 and Yumi No. 3**.

Simple sequence repeats are one of the most informative and versatile molecular markers, which are widely used in genetic diversity, genetic structure and genetic mapping studies (Varshney et al., 2005a,b; Arora et al., 2014; Ting et al., 2014). To provide useful information for marker development in broomcorn millet, we firstly investigated the EST-SSR loci using the assembled transcripts. A total of 35,216 SSR loci were identified in 27,055 sequences out of 113,643 unigenes. Among them, 2,536 sequences contained more than one SSR (**Table 1**). The repeat number of the SSRs ranged from 4 times to 24 times, and a repeat number of four was the most abundant repeats number accounting for 49.99% of SSRs, followed by repeat number of five accounting for 14.45% of SSRs, repeat number of 10 accounted for 13.38% of SSRs, repeat number six accounted for 8.79% of SSRs, while repeat number of more than 20 were rare, only accounted for 0.05% of SSRs. Within the different types of SSRs, trinucleotide was the most abundant repeat unit accounted for 66.72% of SSRs, which was consistent with cabbage, red clover, sweetpotato and cucumber (Guo et al., 2010; Wang et al., 2010; Izzah et al., 2014; Yates et al., 2014), followed by mononucleotide, which accounted for 18.26% of SSRs, dinucleotide, which accounted for 10.33% of SSRs, tetranucleotide, which accounted for 3.15% of SSRs, pentanucleotide, which accounted for 1.24% of SSRs and hexanucleotide accounted for 0.30% of SSRs (**Figure 5**). The most abundant repeat type was A/T, which accounted for 95.88% of the mononucleotide SSRs. The least repeat type was C/G, which accounted for 4.12% of the mononucleotide SSRs. Within dinucleotide repeats, the most abundant motif was AG/CT, which accounted for 62.56% of the dinucleotide SSRs, which was similar with the previous results in sweet potato, coffee, peanut, and Arachis (Poncet et al., 2006; Liang et al., 2009; Wang et al., 2010). In contrast, AC/GT were the most abundant dinucleotide repeats in soybean, maize, rice, wheat, and barley where AC/GT were the most frequent repeats. Within trinucleotide SSRs, CCG/CGG were the most abundant motifs accounting for 46.67% of the trinucleotide SSRs, which were the most abundant trinucleotide repeats in soybean (Xin et al., 2012), maize and barley(Kantety et al., 2002). Previous studies have reported that the CCG/CGG motif was very rare in dicotyledonous plants while abundant in monocots (Wang et al., 2011). The result of Broomcorn millet was consistent with this conclusion (**Table 1**). Finally, the length of SSRs loci were found to range from 10 to 25 bp, of which 12 bp were the most frequent accounting for 51.39% of the SSRs loci, followed by 15 bp accounting for 13.78% of the SSRs loci and 10 bp accounting for 11.91% of the SSRs loci (Supplementary Figure S4).

**Table 1:** Simple sequence repeats (SSRs) identification in broomcorn millet.

Type	Number of SSRs	Repeat in each unit	Number	Percent (%)
Mononucleotide motifs	6,433	A/T	6,167	95.88
		C/G	266	4.12
Dinucleotide motifs	3,635	AG/CT	2,274	62.56
		AC/GT	576	15.84
		AT/AT	412	11.34
		CG/CG	373	10.26
Trinucleotide motifs	23,499	CCG/CGG	10,970	46.67
		AGC/GCT	3,541	15.07
		AGG/CCT	3,182	13.54
		AAG/CTT	1,560	6.64
		ACC/GGT	1,351	5.75
		ACG/CGT	1,180	5.02
Tetranucleotide motifs	1,107	ATTT/AAAT	93	8.40
		AAAG/CTTT	92	8.31
		CCGG/CCGG	60	5.42
Pentanucleotide motifs	436	CCCTT/AAGGG	62	14.22
		GAGGT/ACCTC	58	13.30
Hexanucleotide motifs	106	CCGGCG/CGCCGG	20	18.87
		ACGCCC/GGGCGT	13	12.26
Total number of sequences examined			113,643
Total size of examined sequences (bp)			164,535,293
Total number of identified SSRs			35,216
Number of SSR containing sequences			27,055
Number of sequences containing more than one SSR			2,536
Number of SSRs present in compound formation			786

**FIGURE 6:**
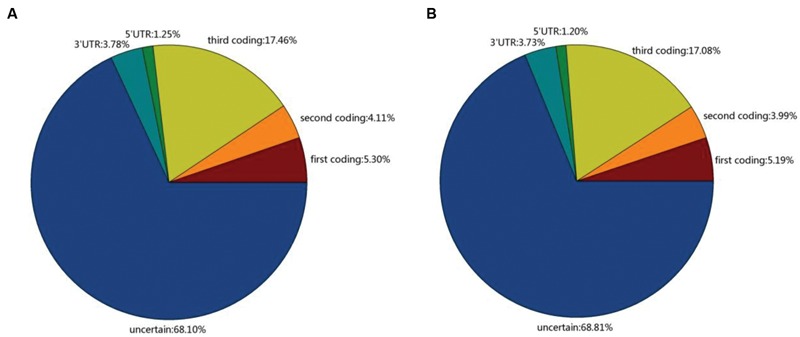
**The distribution of SNPs in Yumi No. 2 **(A)** and Yumi No. 3 **(B)**.** The transcript with no homologous in NR, Swiss-Prot protein database and also no predicted protein-coding potential were identified as non-coding regions.

Single nucleotide polymorphisms have recently become a more popular marker for high density genetic mapping, association mapping and population genetic structure studies (Liu et al., 2011). SNPs occurring in coding regions (cSNP) may cause the loss or change of protein function, and thus, they could be used directly to assess the impact of mutation on important economic traits (Ellegren, 2008). To understand the cSNP in Broomcorn millet, we identified cSNPs between these two cultivars. A total of 406,062 high-quality SNPs were identified in Yumi No. 2 and 409,850 high-quality SNPs were identified in Yumi No. 3. For Yumi No. 2, the putative SNPs included 270,068 transitions (A/G, C/T) and 135,994 transversions (G/T, C/G, A/T, A/C), while 273,593 transitions and 136,257 transversions were observed in Yumi No. 3 (Supplementary Figure S5). The average number was 2.46 and 2.49 SNPs per kb for Yumi No. 2 and Yumi No. 3, respectively. Further analysis found that in Yumi No. 2, 109,078 (26.87%) SNPs were distributed into coding sequences, 20,446 (5.03%) SNPs distributed into untranslated regions (UTRs) and 276,539 (68.10%) distributed in non-coding regions, respectively. Among the SNP of Yumi No. 2, the percentage distributed in the coding sequences (CDSs), UTRs and non-coding regions was 26.87, 5.03, and 68.10%, respectively. However, among the SNP of Yumi No. 3, the percentage distributed in the CDSs, UTRs and non-coding regions was 26.26, 4.93, and 68.81%, respectively (**Figure 6**).

### Detection of Differentially Expressed Genes (DEGs)

**FIGURE 7:**
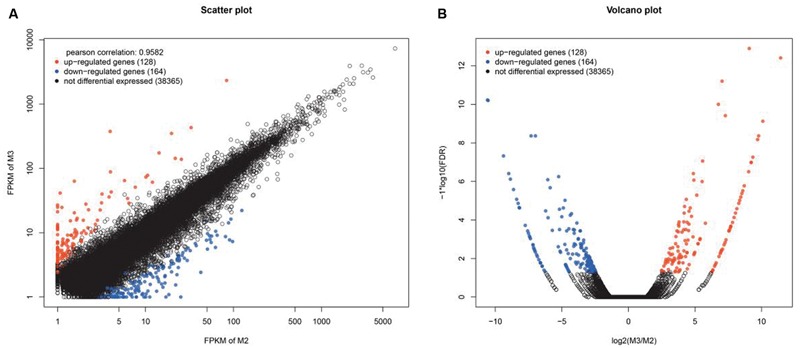
**Scatter **(A)** and Volcano plot **(B)** of the differentially expressed genes (DEGs) between Yumi No. 2 and Yumi No. 3. (A)** The x-axis represents the value of logeRPKM of Yumi No. 2, and the y-axis shows the value of logeRPKM of Yumi No. 3. The black dots represent no significant differences of genes, red and blue dots indicate significantly up-regulated or down-regulated expression of genes in Yumi No. 2 compared to Yumi No. 3 (FDR ≤ 0.001 and log_2_FC ratio ≥ 1), respectively. **(B)** The x-axis respresents the values of log_2_FC for genes of being differentially expressed between Yumi No. 2 and Yumi No. 3. The y-axis shows the values of log_10_FDR. The black dots represent no significant differences of genes, red and blue dots indicate significantly up-regulated or down-regulated expression of genes in Yumi No. 2 compared to Yumi No. 3 (FDR ≤ 0.001 and log_2_FC ratio ≥ 1).

To detect DEGs, the expression level of these unigenes in the two cultivars was investigated. Pairwise comparison of RPKM and FC between Yumi No. 2 and Yumi No. 3 in the RNA-seq data sets was first conducted. The scatter plots showed the expression differences of each gene, and volcano plots showed the expression differences of gene among these two cultivars. The results indicated that most genes were expressed at similar levels (black dots) between the cultivars and only a small portion of genes were significantly up-regulated (red dots) and down-regulated (blue dots) expressed. A total of 292 DEGs were obtained, of which 128 genes (red dots) were up-regulated and 164 genes (blue dots) were down-regulated in Yumi No. 2 compared to Yumi No. 3, all of which had statistically significant differences in expression levels (*P* < 0.05; **Figure 7**). The list of DEGs has shown in Supplementary Table S4 and Supplementary Figure S6.

To determine the potential function of these DEGs, GO, and KEGG analyses were performed. In total, 88 GO terms were significantly enriched in the DEGs at the stringent cut off level of *P* < 0.05. In biological process, the GO terms metabolic process (GO:0008152) and cellular process (GO:0009987) were enriched in the DEGs while in cellular component, the GO terms cytoplasmic part (GO:0044444) and intracellular organelle (GO:0043229) were enriched. Finally, heterocyclic compound binding (GO:1901363) and organic cyclic compound binding (GO:0097159) were enriched in the molecular function category (Supplementary Table S5). KEGG pathway enrichment analysis for DEGs included 12 enriched pathways (Supplementary Table S6). RNA degradation (ko03018), nucleotide excision repair (ko03420), ubiquinone and other terpenoid-quinone biosynthesis (ko00130) and phosphatidylinositol signaling system (ko04070) were the top four enriched pathways. The differential expressed genes clustered into different functional categories, which provided the important resource to discover and identify the important functional genes.

### Validation of the DEGs Using qRT-PCR

**FIGURE 8:**
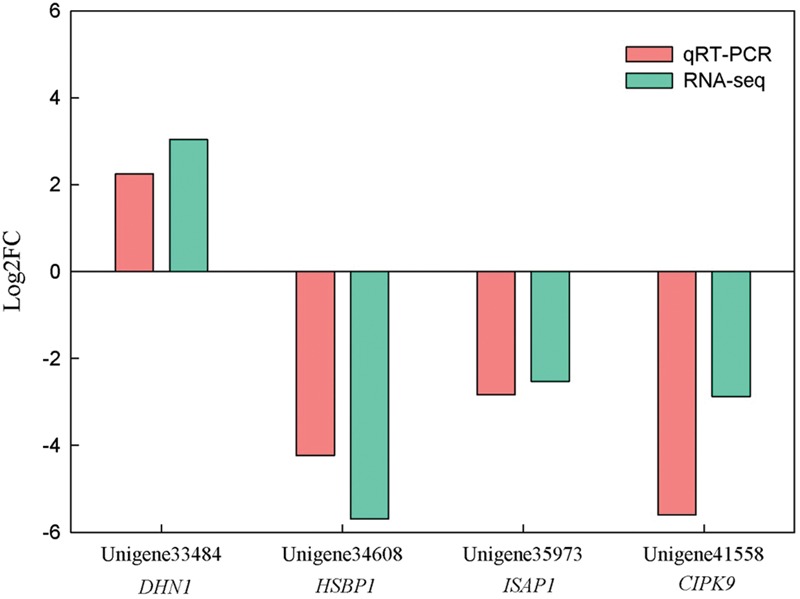
**Expression patterns of four unigenes measured in Yumi No. 2 compared to Yumi No. 3 by RNA-seq and qRT-PCR Analysis.** The x-axis represented the four DEGs, and the y-axis represented the values of log_2_FC. Values were normalized and the proportional fold change (FC) was calculated. Each reaction was performed in triplicate. Pink refers to qRT-PCR, and green to RNA-Seq.

To validate the DEGs from the RNA-seq data, four DEGs which may be involved in abiotic stress response were selected to qRT-PCR analysis. Three DEGs, including *unigene34608, unigene35973* and *unigene41558*, were down-regualted in Yumi No. 2 compared to Yumi No. 3. Unigene33484 was up-regualted in Yumi No. 2 compared to Yumi No. 3. Results showed that the expression patterns of three transcripts (*unigene33484, unigene34608*, and *unigene35973*) were consistent with that of RNA-seq (**Figure 8**). Although, the expression level of the remaining transcript *unigene41558* obtained from qRT-PCR was significantly higher than that of RNA-seq data, they showed a similar down-regulated expression trend to RNA-seq (**Figure 8**). Consequently, the RNA-seq could provide the useful information for gene expression and the identified DEGs provided a valuable resource for gene discovery and functional analysis in Broomcorn millet.

**FIGURE 9:**
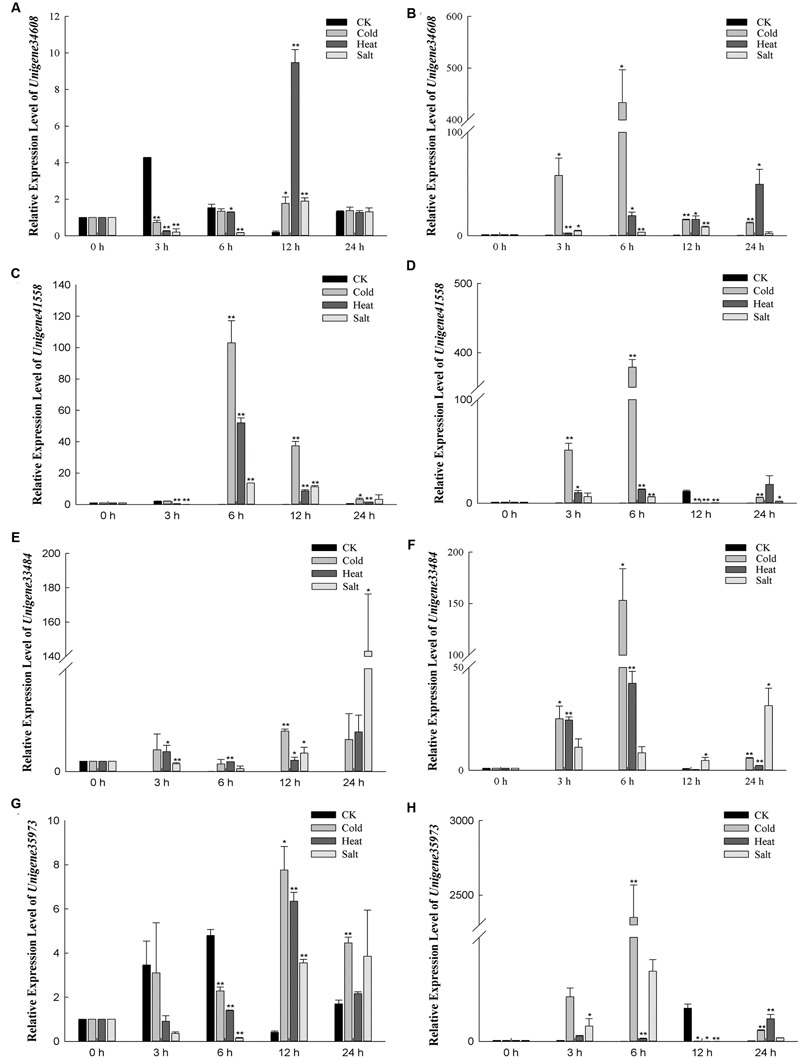
**Relative expression levels of four DEGs which may be involved in abiotic-stress response in Yumi No. 2 and YumiNo. 3 by qRT-PCR analysis.** The 2^-ΔΔCt^ values were used to analyze differential expression levels after cold, heat, salt treatment. Error bars indicate SD (*n* = 3). ^∗^*P*-value < 0.05, ^∗∗^*P*-value < 0.01 (Student’s *t*-test). **(A)** The expression level of *Unigene34608* in Yumi No. 2. **(B)** The expression level of *Unigene34608* in Yumi No. 3. **(C)** The expression level of *Unigene41558* in Yumi No. 2. **(D)** The expression level of *Unigene41558* in Yumi No. 3. **(E)** The expression level of *Unigene33484* in Yumi No. 2. **(F)** The expression level of *Unigene33484* in Yumi No. 3. **(G)** The expression level of *Unigene35973* in Yumi No. 2. **(H)** The expression level of *Unigene35973* in Yumi No. 3.

To identify some stress-related genes from Broomcorn millet, the expression profiles of these unigenes which may be involved in abiotic-stress response were further detected under different stresses (**Figure 9**). *Unigene34608* is predicted to encode heat shock factor-binding protein 1 (HSBP1). HSBP1 can affects HSF1 DNA binding activity and is a negative regulator in response to heat stress (Satyal et al., 1998). In Yumi No. 2, the results showed that the transcript levels of *Unigene34608* had small expression level changes under cold stress, while its expression level was reduced by 0.26-fold under heat stress for 3 h and 0.16-fold after 6 h under salt stress compared to control plants. In Yumi No. 3, the expression level of *Unigene34608* was temporarily elevated under cold and heat stress, especially expression level was increased more than 400-fold compared to control plants under cold treatment for 6 h. And it quickly declined to low levels under salt stress for 24 h. *Unigene41558* putative encodes a CBL-interacting protein kinase 9 (CIPK9), which interacts with calcium sensor and plays important roles in low-K^+^ stress (Liu L.L. et al., 2012; Hung et al., 2014). High expression levels of *Unigene41558* were observed under several stress treatments. For example, in Yumi No. 2, expression levels of *Unigene41558* increased 103.02-fold compared to untreated controls under cold treatment for 6 h, 52.02-fold under heat treatment for 6 h and 11.39-fold under salt treatment for 12 h. Similar trends were also observed in Yumi No. 3 where highest expression level of *unigene41558* were observed under cold stress for 6 h, heat stress for 24 h, and salt stress for 3 h. *Unigene33484* is homologous to an acidic Y2Kn dehydrin DHN1 (Allagulova et al., 2003). A previous study revealed over-expression of *DHN1* gene positively affected plant growth under abiotic stress (Beck et al., 2007). The expression levels of *Unigene33484* show slight increases in expression levels under cold and heat stress in Yumi No. 2 with values ranging from 1- to 4-fold higher than untreated controls, while under salt stress, expression levels initially declined to0.26-fold at6 h and gradually increased to 1.80-fold at 12 h and reached the highest expression levels for 24 h with expression levels 100-fold higher than untreated controls. In Yumi No. 3, the expression patterns of *Unigene33484* under stress treatment were different from Yumi No. 2, which increased 153.22-, 42.27-, 31.38-fold under cold treatment for 6 h, heat stress for 6 h and salt stress for 24 h, respectively. It is indicated that *Unigene33484* likely plays a role in osmoregulation in Broomcorn millet. *Unigene35973* is predicted to encode a zinc-finger protein gene *ISAP1*, which involved in regulating cold, dehydration, and salt tolerance in transgenic tobacco (Mukhopadhyay et al., 2004). In this study, expression levels of *Unigene35973* in Yumi No 2 were 7.72-, 6.34-, 3.75-fold higher under cold, heat, and salt stress compared to untreated controls, respectively. In Yumi No. 3, expression levels of this unigene were over 100-fold higher than untreated controls under both cold and salt stress. The expression profiles of these four unigenes suggested they may play an important role in response to abiotic stress in Broomcorn millet, which provided the foundation for further study of the molecular mechanism of stress tolerance of this important crop.

## Conclusion

This is the first large scale *de novo* assembly and analysis of the transcriptome in broomcorn millet. A total of 113,643 unigenes were obtained, of which 62,543 were functionally annotated. Furthermore, more than 35,000 SSRs and 406,000 SNP loci were identified, which provide an important resource for marker development in this species. This study provided the first insight into the transcriptome of Broomcorn millet, which not only provided an invaluable sequence resource and genomic information for molecular studies in this important crop, but also shed light on discovering the vital functional genes involving in the metabolism regulation network of involved in the adaptation to extreme climatic conditions as well as facilitating further studies on molecular mechanisms of stress tolerance in Broomcorn millet.

## Author Contributions

HY, WS, and XN conceived and designed the experiments. HY and LW performed the experiments. HY, WY, and HL analyzed the data. XD contributed reagents, materials, and analytical tools. HY, WS, and XN wrote the paper. All authors read and approved the final manuscript.

## Conflict of Interest Statement

The authors declare that the research was conducted in the absence of any commercial or financial relationships that could be construed as a potential conflict of interest.

The reviewer ES and handling Editor declared their shared affiliation, and the handling Editor states that the process nevertheless met the standards of a fair and objective review.

## Footnotes

1 http://trinityrnaseq.sourceforge.net

2 https://phytozome.jgi.doe.gov/pz/portal.html#!info?alias=Org_Phallii

3 https://phytozome.jgi.doe.gov/pz/portal.html#!info?alias=Org_Pvirgatum

4 http://foxtailmillet.genomics.org.cn/page/species/download.jsp

5 https://github.com/tanghaibao/Goatools

6 http://pgrc.ipk-gatersleben.de/misa/

7 http://www.bioconductor.org/packages/release/bioc/html/edgeR.html

8 https://cabig.nci.nih.gov/community/tools/geWorkbench

9 https://pypi.python.org/pypi/hcluster/
