# Supplementary material for: De novo Assembly and Characterization of the Transcriptome of Broomcorn Millet (Panicum miliaceum L.) for Gene Discovery and Marker Development
Source: Front Plant Sci. 2016 Jul 21;7:1083. doi: 10.3389/fpls.2016.01083 (PMC4955294; doi:10.3389/fpls.2016.01083)
Supplement: Supplementary file 1 [file Data_Sheet_1.DOCX]

**Supplementary materials**

**De novo** **assembly and characterization of the** **transcriptome of** **Broomcorn millet (*****Panicum miliaceum* L*.*) for gene discovery and marker development**

Hong Yue^1#^, Le Wang^1#^, Hui Liu^1^, Wenjie Yue^1^,Xianghong Du^1^, Weining Song^1,2,3*^Xiaojun Nie^[[1]](#footnote-1),2*^

Corresponding author:

Weining Song (sweining2002@yahoo.com); Xiaojun Nie (small@nwsuaf.edu.cn)


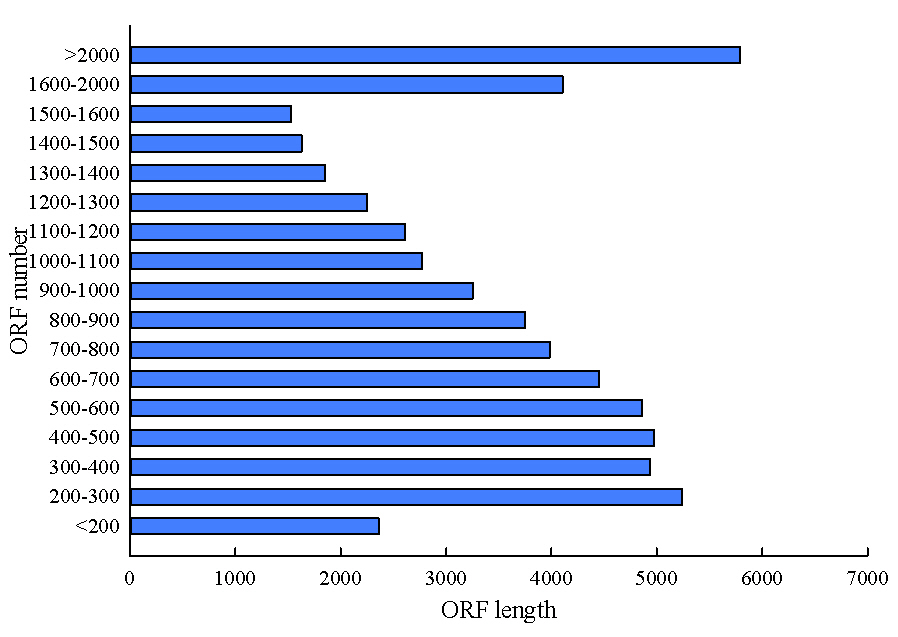


Figure S1. Size distribution of broomcorn millet ORFs


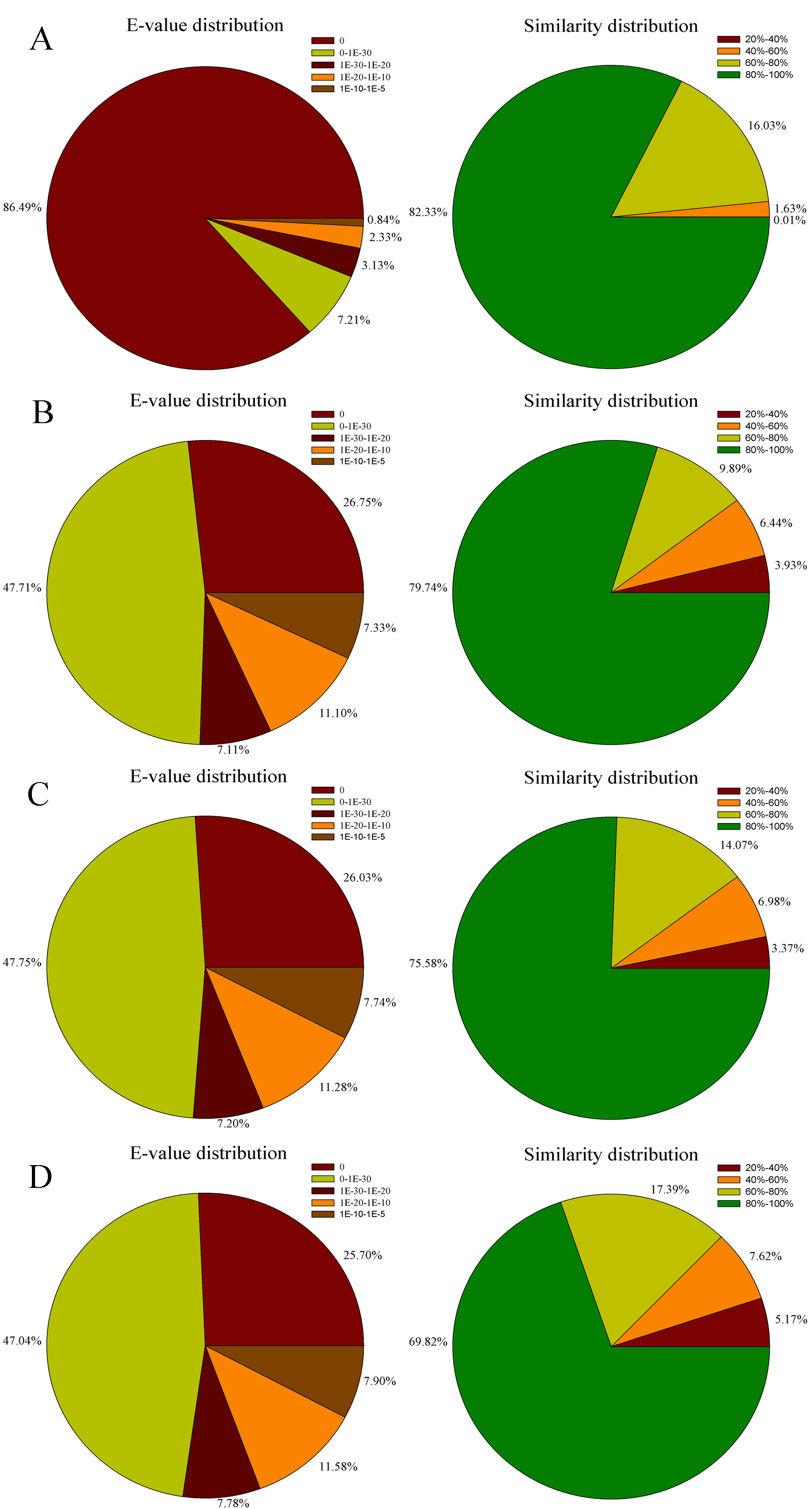


Figure S2. Through against Nr databases, *Panicum halli*, *Panicum virgatum* and *Setaria italica* genome annotations to identify similarity search of transcripts. (A): e-value and percent amino acid similarity from blastx searches against Nr database. (B): e-value and percent amino acid similarity from blastx searches against *Panicum halli* genome annotations. (C): e-value and percent amino acid similarity from blastx searches against *Panicum virgatum* genome annotations. (D): e-value and percent amino acid similarity from blastx searches against *Setaria italica* genome annotations.


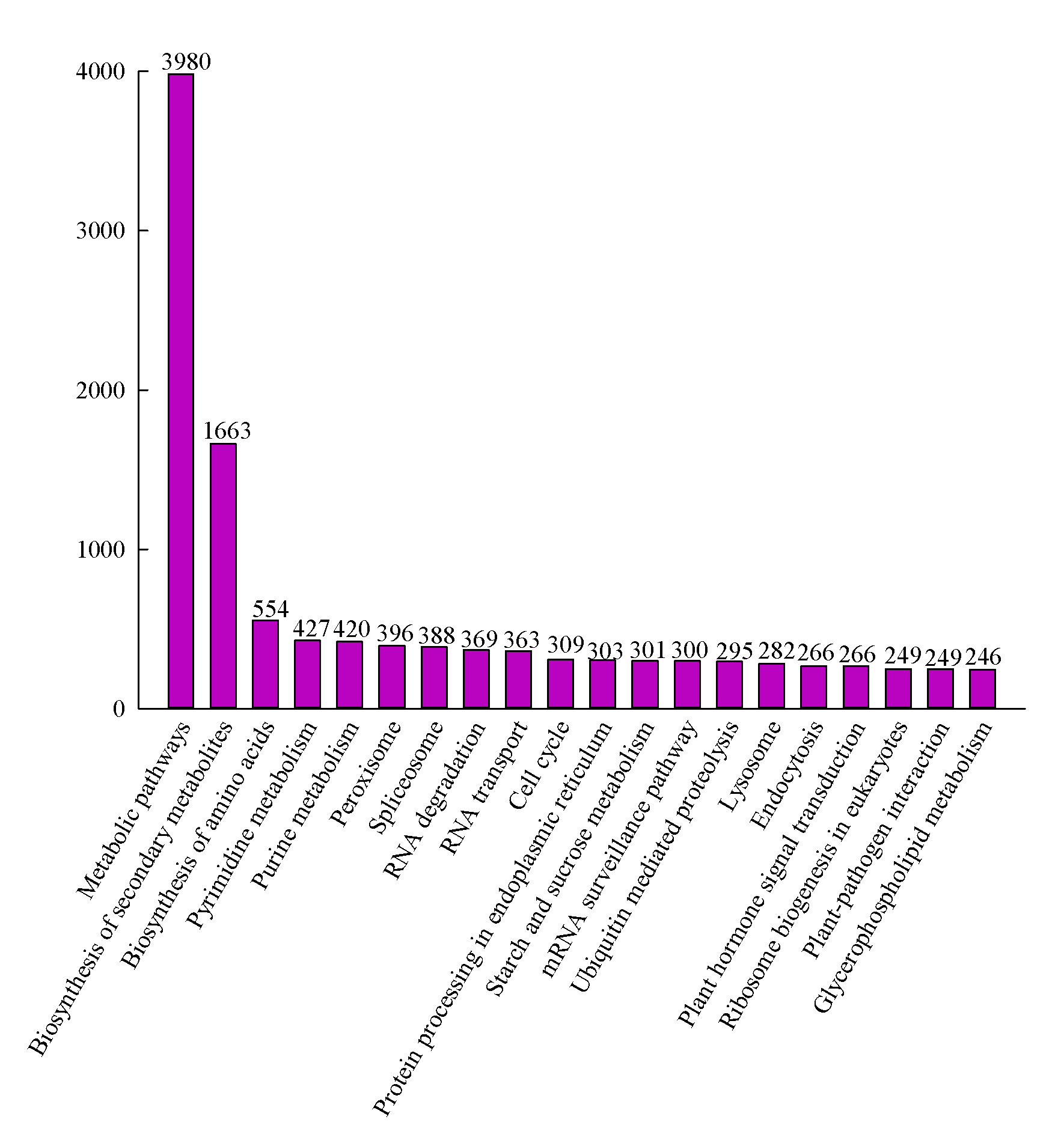


Figure S3. Histogram of the top 20 pathways represented by the unigenes


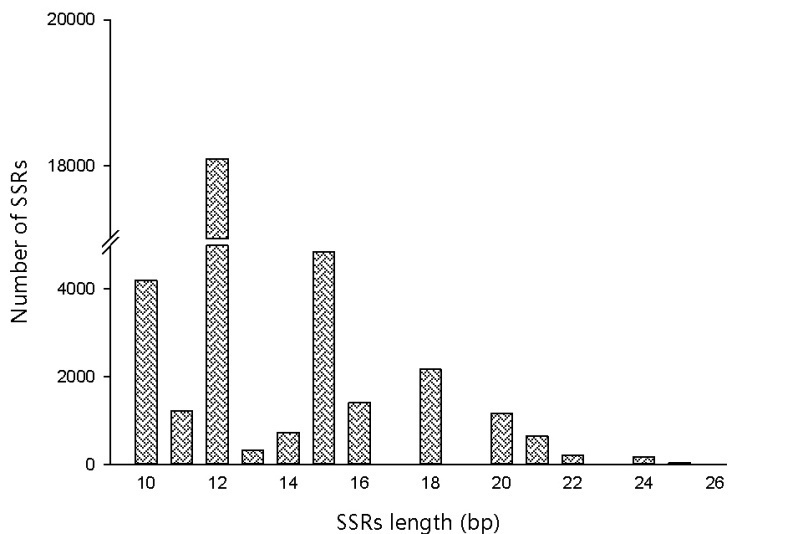


Figure S4. Distribution of different SSRs repeat motifs in Yumi No.2 and Yumi No.3.The x-axis shows total nucleotide length of SSRs, and the y-axis shows number of SSRs


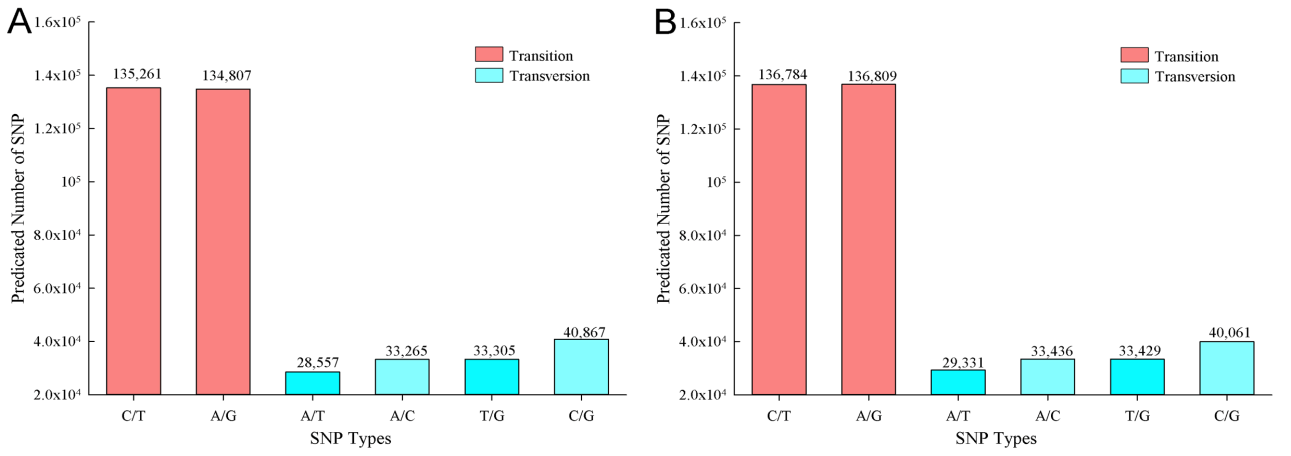


Figure S5. Analysis of single-nucleotide polymorphism (SNP) types in the two common millet cultivars transcriptome.(A): The x-axis shows transitions (pink) and transversions (blue) of Yumi No.2, and the y-axis shows the number of SSR motif. (B): The x-axis shows transitions (pink) and transversions (blue) of Yumi No.3, and the y-axis shows the number of SSR motifs.


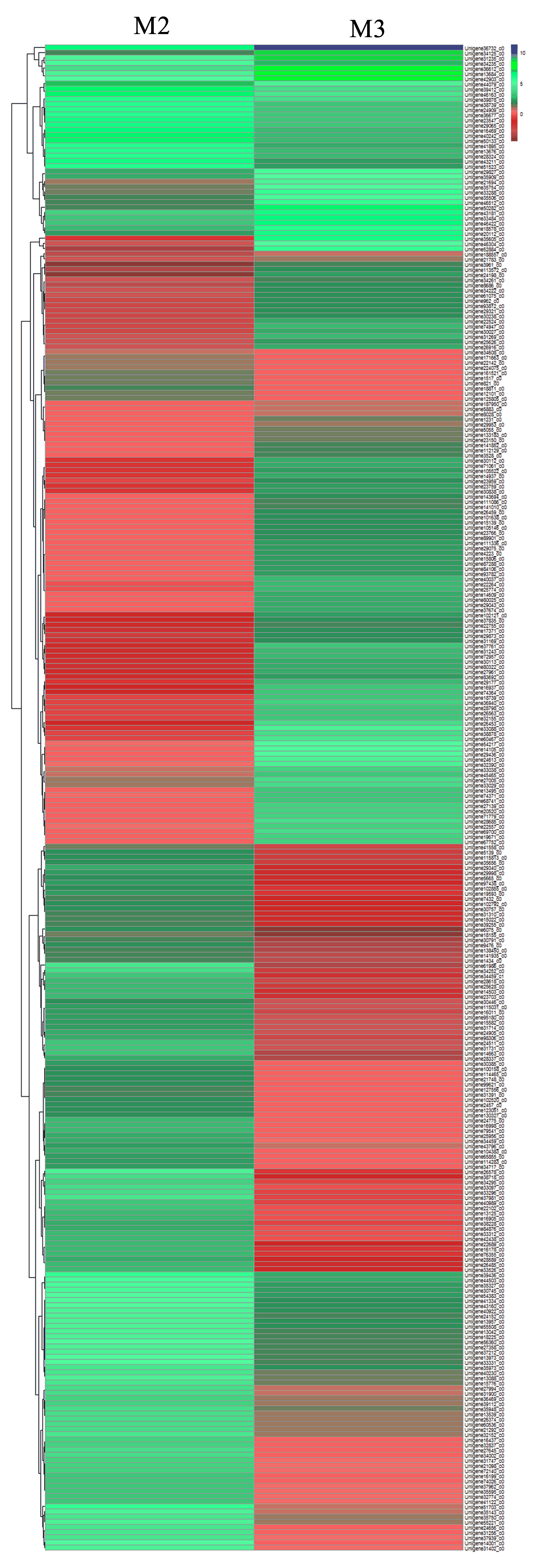


**Figure S6**. Analysis gene expression levels among 2 varieties. The heatmap showed the gene expression variations of 292 DEGs between two varieties. The log_2_RPKM were used to evaluate difference expression levels of genes,and results were displayed with different color intensity. The red, green and black colors representedthe unigene that was down-regulated, up-regulated and significantly up-regulated, respectively.

Table S1. Primers used for quantitative real-time PCR

| Gene ID | Length(bp) | Gene_description | Prime(F/R) |
| --- | --- | --- | --- |
| Unigene33484 | 858 | Setariaitalica DHN1-like, mRNA | F:5’-AACAGGCGGTATCCTGCAC-3’  R: 5’-CCTCCAGGCAATTTCTCCTT-3’ |
| Unigene34608 | 649 | Zea mays heat shock factor-binding protein, mRNA | F:5’-GGATTCAGAGCCGTCATCA-3’  R: 5’-GGTTCCCATTTCATCTATCTTCG-3’ |
| Unigene35973 | 1058 | zinc finger stress-associated protein, mRNA | F:5’-CCCATCATTACACTATTCTTTGTCC-3’  R: 5’-AGAGGAAACACGCATCCAC-3’ |
| Unigene41558  Actin | 1539  171 | CBL-interacting protein kinase, mRNA  Actin, internal gene | F: 5’-AAAGAGGATGGGCTTCTGC-3’  R: 5’-ACAAATAGGATTATGCCACAGG-3’  R: 5’-ACCGAAGCCCCTCTTAACCC-3’  F: 5’-GTATGGCTGACACCATCACC-3’ |

Table S2. Summary of raw reads and clean reads of RNA-seq

| Sample name | Raw reads | Raw reads length (bp) | ≥Q20 (%) | Clean reads | Clean reads length (bp) | ≥Q20 (%) |
| --- | --- | --- | --- | --- | --- | --- |
| Yumi No.2  Yumi No.3 | 47594540  53521908 | 4807048540  5405712708 | 93.93  94.14 | 45406730  51160820 | 4393828933  4961999173 | 98.63  98.65 |

Q20 percentage column shows the proportion of the reads were truncated at any site receiving an average quality score <20 across a 10 bp sliding window.

1. [↑](#footnote-ref-1)
